# Effects of Raloxifene on the Proliferation and Apoptosis of Human Aortic Valve Interstitial Cells

**DOI:** 10.1155/2016/5473204

**Published:** 2016-11-23

**Authors:** Zhimin Fu, Bin Luo, Mingpeng Li, Bin Peng, Zheng Wang

**Affiliations:** ^1^Department of Cardiothoracic Surgery, The First Affiliated Hospital of Jinan University, Guangzhou Shi, China; ^2^Department of Cardiothoracic Surgery, Chenzhou First People's Hospital of Hunan Province, Chenzhou 423000, China; ^3^Department of Cardiac Surgery, Shenzhen People's Hospital, Shenzhen 518020, China; ^4^Cardiovascular Department, Chenzhou First People's Hospital of Hunan Province, Chenzhou 423000, China; ^5^Department of Cardiothoracic Surgery, The 2nd Clinical Medical College of Jinan University, Shenzhen 518020, China; ^6^Department of Cardiothoracic Surgery, Shenzhen People's Hospital, Shenzhen 518020, China

## Abstract

We aimed to explore the effects of raloxifene (RAL) on the proliferation and apoptosis of human aortic valve interstitial cells (AVICs). Different concentrations of RAL were used to act on AVICs. MTS kit is used to test the effects of different concentrations of RAL on the proliferation of AVICs. Cell cycle and apoptosis test used flow cytometry after seven-day treatment. The relative expression levels of caspase-3 and caspase-8 are tested with RT-qPCR and Western blot. The results of MTS testing revealed that the absorbance value (OD value) of the cells in the concentration groups of 10 and 100 nmol/L RAL at a wavelength of 490 nm at five, seven, and nine days significantly decreased compared with that in the control group. Meanwhile, the results of flow cytometry of the cells collected after seven days showed that the ratio of the S stage and the cell apoptosis rate of AVICs can be significantly reduced by RAL in the concentration groups of 10 and 100 nmol/L. The mRNA and protein expressions of caspase-3 and caspase-8 were significantly decreased compared with those in the control group. This study laid the foundation for further treatment of aortic valve disease by using RAL.

## 1. Introduction

The incidence of valvular heart diseases increases yearly with the increase in the population of the elderly, and aortic valve disease accounts for a large proportion of valvular heart diseases [1, 2]. AVICs are the primary structural components that comprise the aortic valves, in which the change of biological function plays an important role in the development of aortic valve disease [3].

RAL belongs to the second generation of selective estrogen receptor modulators (SERMs), which exhibits estrogen-like effects on cardiovascular and bone tissues and antiestrogen effects on uterine and breast tissues with significant tissue selectivity [4]. RAL induces cell death associated with autophagy; the mechanism was mediated by the activation of AMP-activated protein kinase (AMPK) pathway via decreases in intracellular ATP in cancer cells. The overactivation of autophagy can lead to cell death maybe one of the important mechanisms of the therapy effect of RAL [5].

Estrogen can be used to regulate the expression of multiple vascular endothelial genes, protecting against or delaying the development of coronary heart disease. The function of vascular endothelial cell is regulated by RAL via estrogen response elements or other pathways to protect vascular endothelial function [6]. RAL is expected to become a potential drug for the treatment of cardiovascular disease [7, 8].

In this study, human AVICs are isolated using collagenase II and in vitro culture is performed. The effects of different concentrations of RAL on the proliferation and apoptosis of AVICs as well as the relevant genes of cell apoptosis are tested to lay the foundation for further studies on the effects of RAL on aortic valve disease.

## 2. Materials and Methods

### 2.1. Primary Culture and Subculture of AVICs

Human aortic valve was drawn from a 45-year-old female patient without valvular heart disease, who received a heart transplant in the Cardiothoracic Surgery Department of Chenzhou No. 1 People's Hospital, with informed consent signed preoperatively. The valve was carefully removed along the root of the aortic valve and taken back to the laboratory under low temperature. The valve was washed with 1,000 U/mL of antibiotic for 30 s and cleansed with 500, 200, and 100 U/mL of antibiotics for 3 min. The valve tissue was placed in a 600 mm culture dish, the cell culture medium containing 600 U/mL collagenase II was added, and the culture dish was placed in an incubator with 5% CO_2_ under 37°C to digest for 15 min. The endothelial cells were scraped from the surface of the valve tissue with a cell scraper, were cut into 1 mm × 1 mm pieces with sterile microscissors, were placed in a 100 mm culture dish containing digestive juice, were transferred into a 100 mL flask, were digested in an incubator with 5% CO_2_ under 37°C for 6 h, were centrifuged to obtain the primary cultured AVICs, and underwent subculture at a ratio of 1 : 3 when 90% degrees of fusion are reached [9].

### 2.2. Test of the Effects of RAL on the Proliferation of AVICs with the MTS Method

AVICs were inoculated into a 96-well plate with 3,000 cells/well, and three parallel duplicate wells were set in each group. After the cells have completely adhered, 0, 0.1, 1, 10, 100, and 1,000 nmol/L RAL were added in turn, where 0 nmol/L RAL was considered the control group. After the drug was added, 20 *μ*L/well MTS reaction solution was added at zero, three, five, seven, and nine days. The culture dish was placed in an incubator with 5% CO_2_ under 37°C to incubate for 2 h. The absorbance value (OD value) was tested at a wavelength of 490 nm with an automatic microplate reader, and the growth curve was traced.

### 2.3. Test of the Influence of RAL on the Cycle of AVICs by Flow Cytometry

AVICs were inoculated into a six-well plate with 2 × 10^4^ cells/well. After the cells have completely adhered, 0, 0.1, 1, 10, 100, and 1,000 nmol/L RAL were added in turn. After seven days of incubation with 5% CO_2_ under 37°C, the cells were collected, rinsed with phosphate-buffered saline, and centrifuged. Then, 1 mL 75% precooled ethanol was added under −20°C, the sample was resuspended and marked with signs, propidium iodide (PI) staining was performed, and the cell cycle was tested with flow cytometry.

### 2.4. Test of the Influence of RAL on the Apoptosis of AVICs by Flow Cytometry

AVICs were inoculated into a six-well plate with 8 × 10^4^ cells/well. After the cells have completely adhered, 0, 0.1, 1, 10, 100, and 1,000 nmol/L RAL were added in turn. After seven days of incubation following apoptosis induced by a serum-free medium, the cells were centrifuged, the supernatant was removed, and 100 *μ*L 1x binding buffer was added to resuspend the cells. Then, 5 *μ*L APC-Annexin-V and 5 *μ*L PI (BD) were added, staining was performed in the dark under room temperature for 15 min, and testing on the machine was performed.

### 2.5. RT-qPCR Analysis

From the results of the cell cycle and apoptosis of AVICs, we employed 10 and 100 nmol/L RAL for the follow-up tests. The relative expression levels of caspase-3 and caspase-8 were tested using RT-qPCR. The TRIzol kit (Invitrogen, Carlsbad, CA, USA) was used to extract the total RNA of AVICs. Absorbance was tested at 260 and 280 nm using a UV spectrophotometer to determine the level and purity of RNA. The PrimeScript RT reagent kit (TaKaRa Biotechnology Co. Ltd., Shanghai, China) was used to complete the reverse transcription reaction, and the 10 *μ*L total system was used for each reaction. The SYBR Premix Ex Taq II kit (TaKaRa Biotechnology Co. Ltd., Shanghai, China) was used to complete RT-qPCR, and the 20 *μ*L system was used for each reaction. The one-step quantitative PCR system (Applied Biosystems, Foster City, CA, USA) was used to complete the PCR amplification. The standardized H-GDPDH reference was consulted for the relative expression levels of caspase-3 and caspase-8, and 2^−ΔΔCt^ was used to represent the data. The differences among the samples were evaluated. The operations of all the kits mentioned previously were based on the instructions provided by the suppliers. The primers of caspase-3, caspase-8, and H-GDPDH are shown in Table 1.

### 2.6. Western Blot Analysis

RIPA was used to extract the total protein of AVICs by taking the same amount of protein to load and incubate the sample in a blocking solution for an hour after electrophoresis to test caspase-3, caspase-8, and *β*-actin protein. The primary antibody was decolorized with TBST under room temperature, washed twice on a shaking table for 10 min each time after diluting with TBST to 1 : 600, and incubated at room temperature for 1 h to 2 h. The primary antibody was washed with TBS once again for 10 min, incubated with a dilution buffer of a secondary antibody (1 : 1,000) marked by horse radish peroxidase under room temperature for 1 h, decolorized with TBST under room temperature, washed three times on a shaking table for 10 min each time, and tested with the ECL luminescence kit.

### 2.7. Statistical Treatment

The SPSS 17.0 statistical software was used to treat all data. The test data were presented as the mean ± SD. Unpaired *t*-test between two groups was used to compare the test groups with different concentrations of RAL and the control group. *p* < 0.05 represented the statistical difference.

## 3. Results

### 3.1. Morphological Observation of AVICs

**Figure 1:**
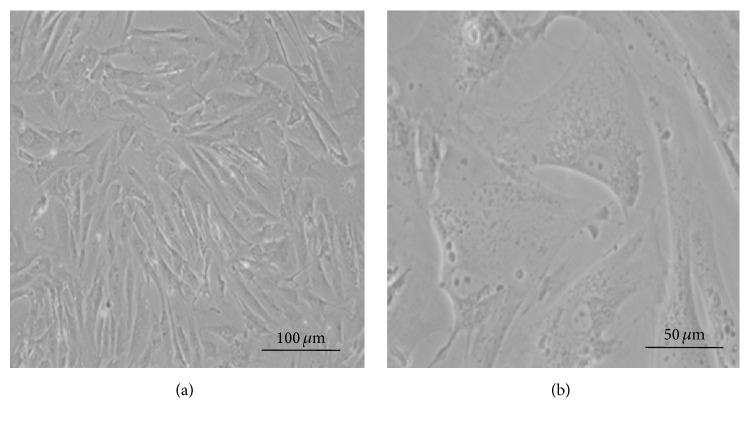
Morphological observation of isolated and cultured human AVICs: (a) ×100 and (b) ×400.

Narrow, strip-like changes were observed after cell attachment of AVICs obtained after isolation that primarily presented as fusiform and polygonal, as shown in Figure 1. The cells grew relatively slow, and the culture medium was replaced once every three days. The subculture was performed every 7 days to 10 days.

### 3.2. Influence of RAL on the Proliferation of AVICs

**Figure 2:**
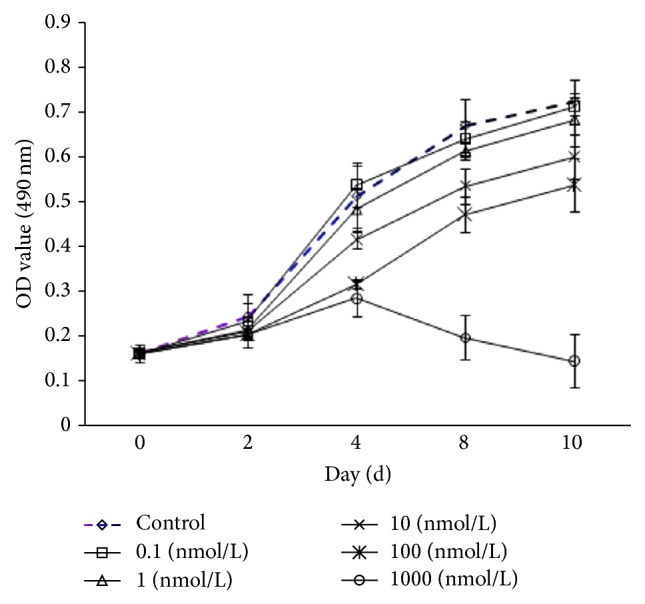
Test of the influence of different concentrations of RAL on the proliferation of AVICs.

The OD values were tested with a microplate reader at a wavelength of 490 nm under different concentrations of RAL at zero, three, five, seven, and nine days. Compared with the control group, the proliferation of AVICs in the test groups was significantly inhibited by 10 and 100 nmol/L RAL at five, seven, and nine days (*p* < 0.05), as shown in Figure 2. A significant inhibition effect of 1,000 nmol/L RAL on the proliferation of AVICs was also observed after five days (*p* < 0.05), as shown in Figure 2. This inhibition effect, which was presented as the OD value, decreased to 0.196  ±  0.029 after seven days when apoptosis of a part of a cell could be observed under an inverted microscope. The OD value further decreased to 0.145 ± 0.017 after nine days when apoptosis of a large number of cells was observed.

**Figure 3:**
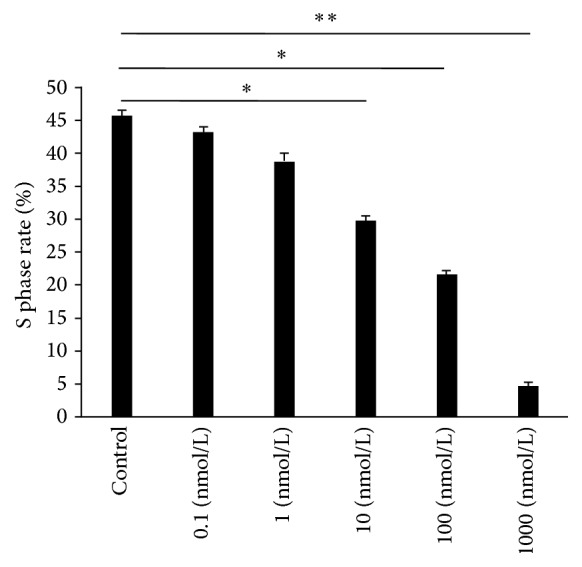
Influence of different concentrations of RAL on the ratio of the S stage of AVICs. The results were presented as the mean ± SD, *n* = 6, ^*∗*^ *p* < 0.05, and ^*∗∗*^ *p* < 0.01.

Tests of the cycle of AVICs with flow cytometry under the action of different concentrations of RAL were performed after seven days, and the following results were obtained. No significant differences in the ratio of the S stage of cells were observed when the 0.1 and 1 nmol/L RAL test groups were compared with the control group. The ratio of the S stage for the 10 nmol/L RAL test group was significantly lower than that of the control group when both groups were compared. By contrast, the ratio of the S stage for the 100 nmol/L RAL test group was also significantly lower than that of the control group when both groups were compared. A statistically significant difference in the ratio of the S stage of cells was observed when the 1,000 nmol/L RAL test group was compared with the control group, as shown in Figure 3. However, combined with the results of MTS testing, the decrease in the ratio of the S stage of cells in the 1,000 nmol/L RAL test group resulted from the apoptosis of AVICs.

### 3.3. Changes of the Apoptosis Rate of AVICs

**Figure 4:**
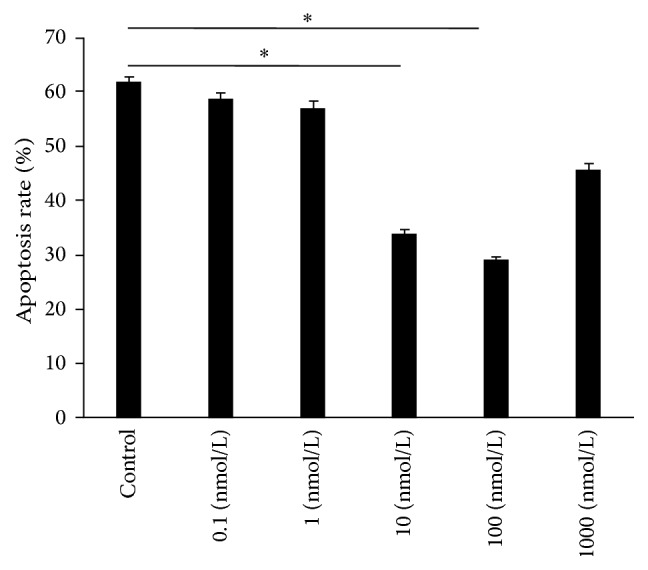
Influence of different concentrations of RAL on the apoptosis rate of AVICs. The results were presented as the mean ± SD, *n* = 6, ^*∗*^ *p* < 0.05, and ^*∗∗*^ *p* < 0.01.

The results revealed that starvation treatment to induce apoptosis was successful, and no differences in the cell apoptosis rate (the sum of the early and late apoptosis rates) were observed when the 0.1, 1, and 1,000 nmol/L RAL test groups were compared with the control group, as shown in Figure 4. The apoptosis rates of AVICs in the 10 and 100 nmol/L RAL test groups were significantly lower than those in the control group when both RAL groups were compared with the control group (Figure 4). Notably, the results of flow cytometry showed that the apoptosis rate of AVICs was 45.43% ± 4.45%. For the 1,000 nmol/L RAL test group, the apoptosis rate of AVICs reached up to 41.26% ± 3.21%.

### 3.4. Influence of Different Concentrations of RAL on the mRNA Expression of Caspase-3 and Caspase-8

**Figure 5:**
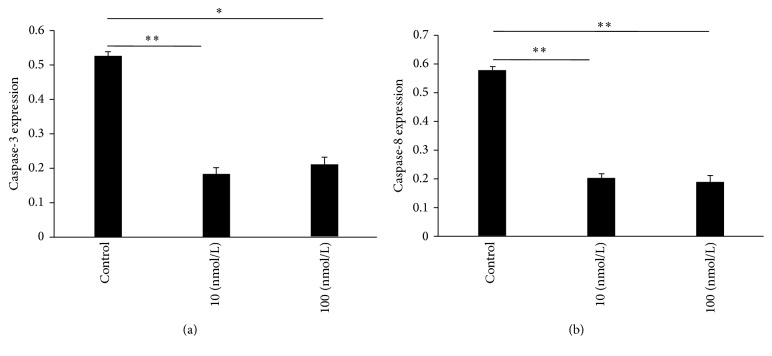
Test of the mRNA expression levels of caspase-3 and caspase-8 at seven days after AVICs were treated with different concentrations of RAL: (a) caspase-3 and (b) caspase-8. The results were presented as the mean ± SD, ^*∗*^ *p* < 0.05, and ^*∗∗*^ *p* < 0.01.

The relative expression levels of the mRNA of caspase-3 and caspase-8 were tested with RT-qPCR. Caspase-3 and caspase-8 are the primary genes of cell apoptosis. The expression levels of both genes were closely related to cell apoptosis. After AVICs were treated with 10 and 100 nmol/L RAL, the mRNA expression levels of caspase-3 and caspase-8 decreased to some extent, and significant differences were observed compared with the control group (Figures 5(a) and 5(b)).

### 3.5. Influence of Different Concentrations of RAL on the Protein Expression of Caspase-3 and Caspase-8

**Figure 6:**
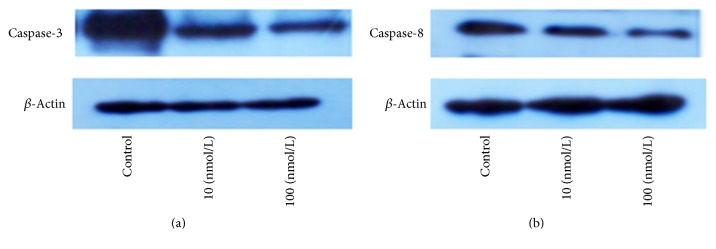
Test of the protein expressions of caspase-3 and caspase-8 at seven days after AVICs were treated with different concentrations of RAL: (a) caspase-3 and (b) caspase-8.

Based on the fact that the expression levels of caspase-3 and caspase-8 were closely related to the apoptosis of AVICs, thus, the protein expression levels of both genes were tested in this study. The results revealed that the protein expressions of caspase-3 and caspase-8 also decreased to some extent at seven days after AVICs were treated with different concentrations of RAL. Significant decreases were observed in the test groups compared with the control group (Figures 6(a) and 6(b)).

## 4. Discussion

Aortic valve disease is a complex pathological process and has been generally considered in the past to be a passive, nonregulated process under the induction of long-term mechanical pressure [10, 11]. However, a series of studies conducted in the past 10 years revealed that aortic valve disease is not only a simple passive process but also an active biological process controlled by active regulation. During the complex regulation process, the proliferation and apoptosis of AVICs were involved in the pathological changes and calcification of valves [12, 13]. Notably, valvular diseases in New Zealand rabbits could be induced by hyperlipidemia as a result of a high-fat diet, whereas the number of apoptotic AVICs in the diseased valve was significantly increased [14, 15]. Jian et al. reported that the calcification of valves could be facilitated via the induction of apoptosis of goat AVICs [16].

As a new generation of SERM, RAL is currently used to treat osteoporosis in females after menopause [17, 18]. RAL has certain effects on cardiovascular diseases, and previous studies revealed that RAL and other SERMs improve the function of vascular endothelial cells [19, 20], dilate the coronary artery [8, 21], and regulate blood fat [22]. However, only a few reports on basic and clinical studies of the effect of RAL on valvular heart disease have been published [23]. Based on the above discussion and description of many literatures, we found that the microsomal antiestrogen-binding site (AEBS) is a high-affinity membranous binding site for the antitumor drug RAL that selectively binds diphenylmethane derivatives of RAL such as PBPE (N-pyrrolidino-4-(phenylmethyphenoxyl)-ethanamine, HCl) and mediates their antiproliferative properties [24–26]. The mechanism of RAL inhibition tumor cell which we more inclined to support through the binding to the AEBS can be involved in the chemopreventive action of SERMs in addition to estrogen receptors.

In the experiments conducted in this study, different concentrations of RAL were applied to act on AVICs, and 10 and 100 nmol/L RAL significantly inhibited the proliferation of AVICs, which indicates that the effects of RAL on AVICs were significantly concentration dependent and were most significant after seven days. Therefore, flow cytometry was applied to test the ratios of the S stage of AVICs under different concentrations to further verify that RAL was concentration dependent. However, with the increase of the drug concentration to 1,000 nmol/L, exfoliation and apoptosis of AVICs were observed, which might be related to the cytotoxic effect resulting from the high drug concentration. Notably, RAL had an antiapoptotic effect on the apoptosis of AVICs induced by serum-free medium, and the effective concentrations were still 10 and 100 nmol/L. However, flow cytometry revealed that when the concentration was 1,000 nmol/L, the total proportion of apoptotic AVICs accounted for more than 98%, which again verified that a high concentration of RAL had certain cytotoxicity. Currently, the apoptosis genes caspase-3 and caspase-8 are considered to be important genes in the regulation of the proliferation and apoptosis of AVICs, which are involved in the physiological and pathological apoptosis processes of AVICs induced by multiple factors. The results of our study showed that certain concentrations of RAL had significant inhibition effects on the expressions of caspase-3 and caspase-8 for AVICs induced by serum-free medium.

Considering the previously presented description, the proliferation and apoptosis of AVICs are involved in the pathological changes and calcification of valves. Therefore, we have reason to believe that, in vivo, the appropriate and effective blood concentration of RAL can potentially alleviate aortic valve disease by inhibiting the proliferation and apoptosis of AVICs.

## Figures and Tables

**Table 1:** Sequences of primers for real-time quantitative PCR.

Gene	Primer sequences
Caspase-3	Forward primers 5′-GGAACAAATGGACCTGTTGACC-3′
	Reverse primers 5′-AGGACTCAAATTCTGTTGCCACC-3′
Caspase-8	Forward primers 5′-AGCAAAGGGGAGGAGTTGTG-3′
	Reverse primers 5′-TACTGTGCAGTCATCGTGGG-3′
H-GDPDH	Forward primers 5′-TGGACCTGACCTGCCGTCTA-3′
	Reverse primers 5′-GCAGTGGGTGTCGCTGTTGA-3′
